# Correlation between PSOGI pathological classification and survival outcomes of patients with pseudomyxoma peritonei treated using cytoreductive surgery and HIPEC: national referral centre experience and literature review

**DOI:** 10.1515/pp-2023-0001

**Published:** 2023-05-01

**Authors:** Lorena Martín-Román, Enda Hannan, Mohammad Faraz Khan, Anna Sophia Müller, Conor Shields, John Aird, Brendan Moran, Jurgen Mulsow

**Affiliations:** Department of Surgery, National Centre for Peritoneal Malignancy, Mater Misericordiae University Hospital, Dublin, Ireland; Department of Pathology, Mater Misericordiae University Hospital, Dublin, Ireland; Peritoneal Malignancy Institute, Basingstoke and North Hampshire Hospital, Basingstoke, UK

**Keywords:** acellular mucin, appendiceal mucinous neoplasms, appendiceal mucinous neoplasms with signet ring cells, cytoreductive surgery and hyperthermic intraperitoneal chemotherapy, grading pathology and classification, pseudomyxoma peritonei

## Abstract

**Objectives:**

The Peritoneal Surface Oncology Group International (PSOGI) consensus subdivided pseudomyxoma peritonei (PMP) into four groups according to histopathological features. The aim of this paper is to report survival outcomes after cytoreductive surgery (CRS) and hyperthermic intraperitoneal chemotherapy (HIPEC) from a national referral centre and to correlate the PSOGI classification with survival.

**Methods:**

A retrospective study of a prospectively maintained database was performed. Consecutive patients treated with CRS + HIPEC for PMP of appendiceal origin were included (September-2013 to December-2021). Pathological features of the peritoneal disease were used to classify patients into the four groups proposed by PSOGI. Survival analysis was performed to evaluate the correlation of pathology on overall survival (OS) and disease-free survival (DFS).

**Results:**

Overall, 104 patients were identified; 29.6 % were reclassified as acellular mucin (AM), 43.9 % as low-grade mucinous carcinoma peritonei (LGMCP), 22.4 % as high-grade MCP (HGMCP) and 4.1 % as HGMCP with signet ring cells (HGMCP-SRC). Median PCI and rate of optimal cytoreduction were 19 and 82.7 %, respectively. Median OS and DFS were not reached, 5-year OS and DFS were 88.6(SD 0.04) % and 61.6(SD 0.06) %, respectively. Log-Rank test revealed significant differences in terms of OS and DFS across the different histological subgroups (p<0.001 in both cases). However, histology did not retain its significance in the multivariate analysis for OS or DFS (p=0.932 and p=0.872, respectively).

**Conclusions:**

Survival outcomes after CRS + HIPEC for PMP are excellent. The PSOGI pathological classification correlates with OS and DFS, but differences were not significant at multivariate analysis when adjusted for other prognostic factors.

## Introduction

Appendiceal mucinous neoplasms form a heterogeneous group of benign and malignant tumours with a predilection towards peritoneal dissemination [1]. The presence of mucin throughout the abdominal cavity with, or without, tumour masses is a clinicopathological condition known as *pseudomyxoma peritonei* (PMP). PMP is uncommon, with an estimated incidence of 1–3 per million people annually [2]. Its clinical course varies from a slow growing indolent neoplasm resulting in intraperitoneal mucin accumulation to an aggressive and invasive malignancy with capacity to metastasize and rapidly limit survival. Even after gold standard treatment by optimal cytoreductive surgery and hyperthermic intraperitoneal chemotherapy (CRS + HIPEC) [3], prognosis is mainly determined by the pathological features of the mucinous peritoneal deposits [4], [5], [6].

In 2016, the consensus led by Peritoneal Surface Oncology Group International (PSOGI) assumed the arduous task of unifying and clarifying the ambiguous terminology surrounding this disease. This resulting classification system defined pathological criteria of both the primary appendix tumour and peritoneal deposits [7]. The terms low-grade appendiceal mucinous neoplasm (LAMN) and high-grade appendiceal mucinous neoplasm (HAMN) were incorporated into the classification of primary lesions. Peritoneal disease was subdivided into the following four categories: acellular mucin (AM), low-grade mucinous carcinoma peritonei (LGMCP), high-grade mucinous carcinoma peritonei (HGMCP), and HGMCP with signet ring cells (HGMCP-SRC), from least to most aggressive. The reproducibility [8], and integration of the PSOGI Classification into recent European clinical guidelines [9], has further expanded its use and helped unify the language used amongst experts.

However, work is ongoing on whether the PSOGI classification system stratifies patients into prognostic outcome groups. Reported results reported have been inconsistent. In 2017, Huang et al. [10] observed that the four-tiered PSOGI classification significantly correlated with survival whereas Baratti et al. [8] were unable to reproduce these results. In order to generate effective treatment and follow-up regimes, both a universal language and adequate patient stratification are needed.

The aim of this study was to correlate the PSOGI classification system with survival and to report survival outcomes in patients with PMP treated by CRS + HIPEC in a national centre. A secondary aim was to compare the results with existing scientific literature.

## Materials and methods

A retrospective study of a prospectively maintained database was analysed. All consecutive patients treated with CRS + HIPEC for PMP at the Peritoneal Malignancy Institute at the Mater Misericordiae University Hospital (MMUH) from September 2013 to December 2021 were included. Medical charts, clinical letters, operative notes, laboratory and histopathology reports were examined. Data was cross-checked against the prospectively maintained database.

The study was evaluated and approved by the Institutional Review Board at the MMUH.

### Patient selection and clinical management

In 2013 a program to provide CRS HIPEC to appropriately selected patients with peritoneal malignancy was established. The program has been delivered at a single national centre and has provided care to patients with pseudomyxoma peritonei from throughout the Republic of Ireland.

The decision to proceed to CRS + HIPEC was at the multidisciplinary meeting (MDT). An extensive clinical history including serum tumor marker status (TM) (carcinoembryonic antigen (CEA), carbohydrate antigen 19-9 (Ca19-9) and cancer antigen 125 (Ca-125)) was recorded. Radiologists with a special interest and experience in peritoneal malignancy reviewed oral and intravenous contrast-enhanced computed-tomography (CT) scans of chest/abdomen/pelvis [11]. An expert pathologist reviewed available tissue (mainly from previous appendicectomy or debulking specimens or radiologically guided biopsies).

Intraoperatively, the extent of the peritoneal disease was recorded using the peritoneal cancer index (PCI) scoring system [12]. Briefly, the abdomen is divided into 9 regions with 4 further regions for the small bowel, resulting in 13 in total. A score of 0–3 is calculated for each region where 0 indicates that no visible peritoneal disease, 1 where tumour nodules are <0.5 cm, 2 being nodules from 0.5 to 5 cm and 3 nodules >5 cm or confluent disease. The PCI score ranges from 0 to 39. The objective of CRS was to remove all macroscopic tumor deposits with HIPEC aiming to treat microscopic disease using a single dose of heated chemotherapy directly in contact with any tumour nodules or presumed residual microscopic disease [13]. Visceral resections were used where needed taking into consideration non-vital organ involvement by disease and the histology of the primary appendix tumour. Thus, a radical appendicectomy would suffice in patients with a LAMN, but a right hemi-colectomy with lymphadenectomy was performed in patients with a HAMN or mucinous adenocarcinomas +/−SRC). Peritonectomy procedures were performed as described by Sugarbaker [14]. The completeness of cytoreduction score (CC) was recorded where CC-0 implies no residual macroscopic disease, CC-1 where residual tumour nodules are <2.5 mm in size, CC-2 from 2.5 mm to 2.5 cm and CC-3 >2.5 cm [12]. For PMP, optimal CRS is considered where patients have had CC0 or CC1 cytoreduction as HIPEC can penetrate and eradicate tumour nodules up to 3 mm in size. After CRS completion, HIPEC was delivered using either the open “coliseum” technique or closed technique for 60 min at 41-43 °C using a delivery circuit (SunChip2, Gamida, France). The dosage protocols were based on the body surface area (mitomycin-C 10 mg/m^2^). Pelvic anastomosis, when needed, was performed after completion of HIPEC. Low pressure abdominal surgical drains were placed routinely. Patients were admitted to a critical care unit for at least 48 h for postoperative monitoring. Parenteral nutrition and mechanical and pharmacological anti-thrombotic prophylaxis were initiated in all cases. Perioperative mortality and complications were recorded using the Clavien–Dindo classification system [15]. All cases were rediscussed at the MDT once pathology results were available. Oncology review was advised for patients with pathological high-grade features. Follow-up varied according to the final pathology but at a minimum occurred every six months during the first year; yearly up until the tenth year. Follow-up by treating surgeon or medical oncologist at the treatment centre involving clinical examination, TM measurement and CT-scan evaluation. The date, site and treatment offered for recurrence was recorded. The date of death (regardless of the cause) was registered.

### Pathological evaluation

The pathology specimens obtained from the intervention after the year 2016 were analyzed by an experienced pathologist (J.A) who classified peritoneal implants using the PSOGI classification [7]. Specimens removed before this period were reviewed and reclassified into the PSOGI classification groups.

Primary appendiceal lesions were categorized into benign lesions, LAMN, HAMN and mucinous adenocarcinoma. Pushing invasion is the main pathological feature that differentiates LAMN and HAMN lesions from mucinous adenocarcinomas which demonstrate an infiltrative growth pattern [16]. Mucinous adenocarcinomas with SRC (w/SRC) and signet ring cell carcinoma (SRCC) were defined by the presence of <50 % and >50 % of SRC respectively. The presence of cells with neuroendocrine differentiation (positive chromogranin/synaptophysin immunohistochemical staining) classified a lesion as Goblet cell carcinoma and was excluded from the current analysis [17].

Peritoneal lesions were classified into the following categories: acellular mucin (AM), low-grade mucinous carcinoma peritonei (LGMCP), high-grade mucinous carcinoma peritonei (HGMCP) and HGMCP with signet ring cells (HGMCP-SRC). AM was defined as mucin and a granulation-like response in the peritoneum with absence of epithelial cells. Mucinous deposits with <20 % of low-grade epithelial cells correspond to LGMCP category, to HGMCP, when >20 % of cells with high-grade features and to HGMCP-SRC when at >10 % of cells are SRC. Cases without mucin or mucinous epithelial cells could not be classified according the PSOGI criteria, and were excluded.

### Statistical analysis

The primary objective was to evaluate OS and DFS in the different histological subgroups. The secondary objective was to evaluate the impact of PCI, CC score and preoperative TM status on outcomes.

The analysis of variance (ANOVA) test and non-parametric tests (Mann-Whitney U test or Kruskal–Wallis test) were used to analyse differences in continuous variables across the different categories. The Pearson’s chi-square was used to compare categorical data. The Kaplan–Meier method was used to perform survival analysis and the log-rank test for group comparisons. OS was considered as the period of time (in months) from the date of CRS/HIPEC to the date of death (regardless of the cause) and DFS, to the date of recurrence in cases with optimal CRS. Patients without events (death or recurrence) were censored at the day of last contact and patients who had postoperative deaths were excluded. Multivariate analysis was performed using a cox-regression model and identified possible confounding variables. Missing data was managed via deletion methods. Statistical significance was defined at p<0.05.

Statistical analysis and data management was done using SPSS version 23.0 (IBM) and R-studio.

## Results

Between September 2013 and December 2021, a total of 104 patients underwent CRS + HIPEC for appendiceal tumours with confirmed or suspected PMP. In 6 cases, no evidence of peritoneal mucin or mucinous epithelial cells was identified during pathologic examination. These 6 cases had CRS + HIPEC for either oncological reasons in patients who had adverse appendiceal pathology at appendicectomy or CT suspected disease. The primary tumour was a LAMN in two, three moderately to poorly differentiated mucinous adenocarcinoma, and one unknown primary appendiceal lesion. However, these cases could not be classified into any of the PSOGI categories and were excluded from the current analysis resulting in a final cohort of 98 patients.

### Characteristics of cohort and overall outcomes

The pathology of the primary appendix tumour was available and documented in 91 patients. Following PSOGI’s description, these were classified into LAMN in 53 (58.2 %); HAMN in 5 (4.8 %), well-differentiated mucinous adenocarcinoma in 14 (15.4 %); moderate-to-poorly differentiated mucinous adenocarcinoma in 14 (15.4 %), mucinous adenocarcinoma with SRC in 3 (3.3 %) and SRCC in 1 (1.1 %). Evaluation of peritoneal implants stratified patients into 29 cases of AM (29.6 %), 43 LGMCP (43.9 %); 22 HGMCP (22.4 %) and 4 HGMCP-SRC (4.1 %). A significant correlation between primary appendiceal lesions and the pathology of the associated peritoneal disease (p<001) was observed. In total, 92 % of AM cases originated from an appendiceal LAMN whereas 94.6 % of LGMCP cases came from a LAMN (64.9 %) or a well-differentiated mucinous adenocarcinoma (29.7 %). HGMCP cases were more frequently associated with a moderate-to-poorly-differentiated mucinous adenocarcinoma (52.6 %) and 75 % of HGMCP-SRC were associated with a mucinous adenocarcinoma with SRC (50 %) or a SRCC (25 %).

The mean age was 58 years and 65/98 (62.5 %) were female. Overall, 20/65 (30.8 %) women were referred after gynecological debulking surgery due to initial suspicion of an ovarian neoplasm. Table 1 summarizes the clinical characteristics of the cohort. The median PCI score was 19 (IQR 9–28) and optimal CRS (CC0/1) was achieved in 82.7 %. The median length of stay (LOS) was 15 days (IQR 12–20) and the rate of severe postoperative complications defined by Clavien–Dindo>3 was 18.3 %. There was one postoperative death from a myocardial infarction. There was no relationship between histological subgroups and postoperative morbidity (p=0.679), nor LOS (p=0.128). The median follow-up was of 30.2 months (IQR 15.8–58.1 months). Three patients were lost to follow-up and 10/95 patients died during the follow period. The 2- and 5-year survival rates of the entire cohort were 92.1% and 88.8 %, respectively. Disease recurrence was detected in 22/77 (28.6 %) patients. The most frequent site of recurrence was peritoneal (in 19, 86.3 %), followed by 3 cases of multisite recurrence (13.6 %). Median DFS was not reached; 2- and 5-year DFS rates were of 79.1% and 61.6 % respectively (see Table 1).

**Table 1:** Characteristics of the cohort.

	Whole series (n=104)	AM (n=29)	LGMCP (n=43)	HGMCP (n=22)	HGMCP-SRC (n=4)	p-Value
**Gender**						p=0.091^a^
Male	39 (37.5)	7 (24.1)	18 (41.8)	11 (50)	0	
Female	65 (62.5)	22 (75.9)	25 (58.1)	11 (50)	4 (100)	
**Age, years**						p=0.112^b^
Mean, SD	58 (12.04)	55.1 (11.5)	59 (12.0)	62.8 (10.5)	54.7 (7.3)	
**ASA score**						p=0.129^a^
1	19 (18.4)	7 (24.1)	3 (7.1)	4 (18.2)	1 (25)	
2	57 (55.3)	14 (48.3)	30 (71.4)	9 (40.9)	3 (75)	
3	27 (26.2)	8 (27.6)	9 (21.4)	9 (40.9)	0	
**PCI**						p<0.001^c^
Median (IQR)	19 (9–28)	11 (6–19)	21 (12–31)	29 (19–38)	19 (15–28)	
**CC score**						p=0.001^a^
0	55 (52.9)	22 (75.9)	18 (41.9)	8 (36.4)	1 (25)	
1	31 (29.8)	7 (24.1)	16 (37.2)	5 (22.7)	3 (75)	
2–3	18 (17.3)	0	9 (20.9)	9 (40.9)	0	
**CEA (>5 ng/mL)**	33/75 (44)	3 (12.5)	20 (66.7)	10 (66.7)	0	p<0.001^a^
**CA 19-9 (>23 UI/mL)**	33/73 (45.2)	3 (16.7)	16 (48.5)	12 (70.6)	1 (50)	p=0.015^a^
**Ca-125 (>35 UI/mL)**	27/59 (45.8)	3 (15.8)	13 (54.2)	11 (90.9)	1 (50)	p<0.001^a^
**PSOGI classification 1° appendiceal lesion**						p<0.001^a^
NA	13					
LAMN	53 (58.2)	23 (92)	24 (64.9)	3 (15.8)	1 (25)	
HAMN	5 (4.8)	1 (4)	1 (2.7)	3 (15.8)	0	
G1 ADC	14 (15.4)	1 (4)	11 (29.7)	2 (10.5)	0	
G2-3 ADC	14 (15.4)	0	1 (2.7)	10 (52.6)	0	
ADC w/SRC	3 (3.3)	0	0	1 (5.3)	2 (50)	
SRCC	1 (1.1)	0	0	0	1 (25)	
**LN**						p<0.001^a^
N0	97 (94.2)	29 (100)	43 (100)	18 (81.8)	1 (33.3)	
N+	6 (5.8)	0	0	4 (18.2)	2 (66.7)	
**Postoperative SCT**	13 (16.5)	0	0	12 (70.6)	1 (33.3)	p<0.001^a^
**DFS**						
Median	NR	NR	NR	25	10.2	p<0.001^d^
2y, SD	79.1 (0.05)	100	78.7 (0.07)	50.3 (0.14)	33.3 (0.27)	
5y, SD	61.6 (0.06)	100	55.8 (0.10)	18.9 (0.15)	0	
**OS**						p<0.001^d^
Median	NR	NR	76.1	NR	NR	
2y, SD	92.1	100	97.4 (0.03)	71.4 (0.11)	66.7 (0.27)	
5y, SD	(0.03)	100	93.6 (0.04)	53.6 (0.18)	66.7 (0.27)	
	88.8					
	(0.04)					

Statistical test: ^a^Chi square test; ^b^ANOVA; ^c^Kruskall-Wallis test; ^d^Log-Rank test. ASA, American Society of Anesthesiologists Classification; SC, systemic chemotherapy; PCI, peritoneal carcinomatosis index; CC, completeness of cytoreduction; HIPEC, hyperthermic intraperitoneal chemotherapy; LOS, length of stay; NA, not available; LAMN, low grade appendiceal mucinous neoplasms; G1 ADC, well-differentiated mucinous adenocarcinoma of the appendix; G2-3 ADC, moderately to poorly differentiated mucinous adenocarcinoma of the appendix; ADC w/SRC, mucinous adenocarcinoma of the appendix with signet ring cells; SRCC, signet ring cell carcinoma; PSOGI, Peritoneal Surface Oncology Group International; AM, acellular mucin; LGMCP, low grade mucinous carcinoma peritonei; HGMCP, high grade mucinous carcinoma peritonei; HGMCP-SRC, high grade mucinous carcinoma peritonei with signet ring cells; LN, lymph node status; HR, Hazard ratio; NS, not significant; NR, not reached.

The distribution of factors such as PCI, CC score, TM status, lymph node metastasis and adjuvant SCT administration was unequal across the different pathological subgroups (see Table 1).

### Correlation between PSOGI classification and survival

**Figure 1:**
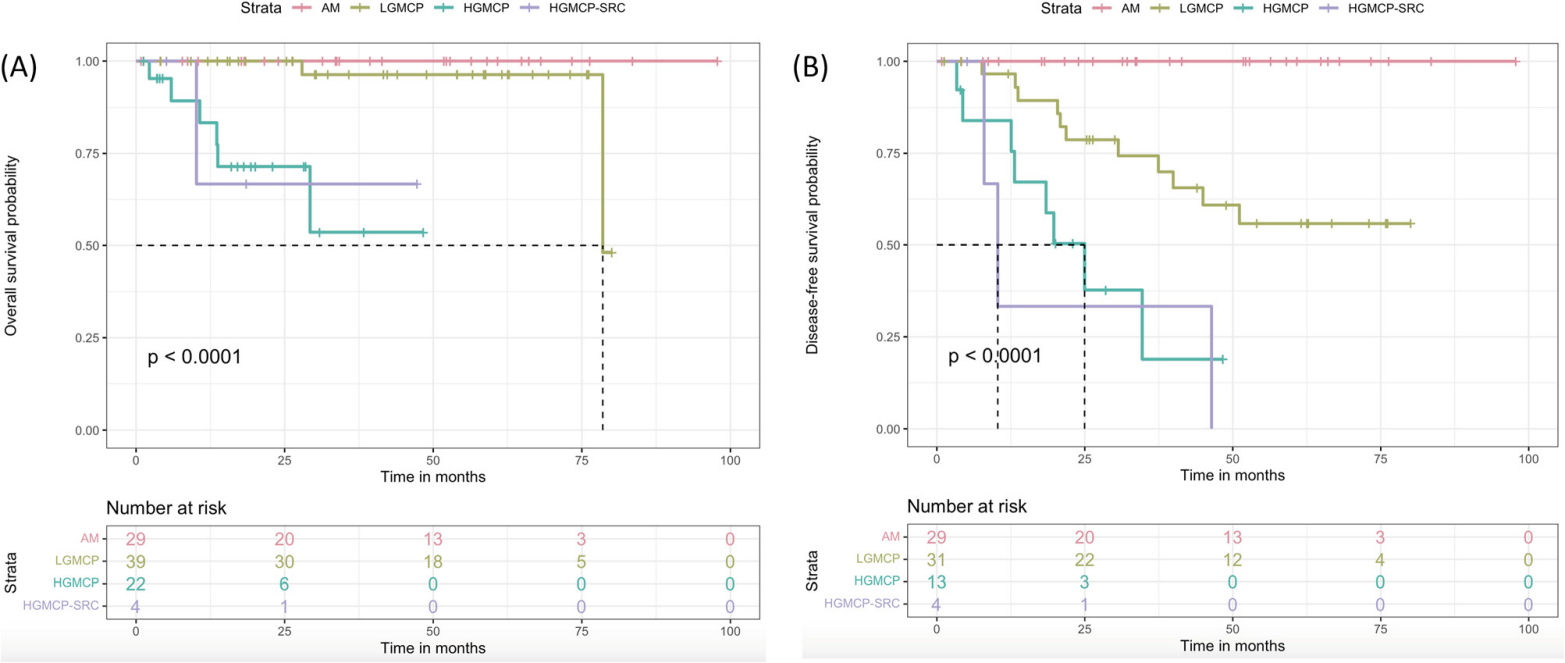
Overall survival (a) and disease-free survival (b) curves according to the PSOGI classification. (A) 5-year overall survival rates of 100 % in AM patients, 96.3 % in LGMCP, 53.6 % in HGMCP and 66.7 % in HGMCP-SRC (p<0.001). (B) 5- year disease-free survival rates of 100 % in AM patients, 55.8 % in LGMCP, 18.9 % in HGMCP and 0 % in HGMCP-SRC (p<0.001).

The PSOGI classification predicted OS in the univariate analysis (see Table 1 and Figure 1A). No deaths were observed in the AM subgroup; 2 in the LGMCP; 6 in the HGMCP and 1 in the HGMCP-SRC subgroup. The 5-year OS rates across the groups were 100 % in AM, 93.6 % in LGMCP, 53.6 % in HGMCP and 66.7 % in HGMCP-SRC (Log-Rank p<0.001). Pairwise comparisons revealed differences to be significant between AM vs. HGMCP (p=0.001) and AM vs. HGMCP-SRC (p=0.004) and LGMCP vs. HGMCP (p=0.003). Median OS was only reached in the LGMCP subgroup (76.1 months, see Figure 1A).

The predictive effect of the PSOGI classification on DFS was also significant on univariate analysis (see Table 1 and Figure 1B). Again, no relapses were observed in the AM group, whereas 11, 8 and 3 were observed in the LGMCP, HGMCP and HGMCP-SRC subgroups, respectively. Recurrences in the LGMCP and HGMCP-SRC subgroups were peritoneal and 3/8 patients in the HGMCP had a multisite recurrence (37.5 %), p=0.048. Median DFS was not reached in AM and LGMCP patients, but was 25 months in HGMCP and 10.2 months in HGMCP-SRC. The 5-year DFS rates were 100 %, 55.8%, 18.9% and 0 % in AM, LGMCP, HGMCP and HGMCP-SRC, respectively. These differences were significant in the Log-Rank test (p<0.001). Pairwise comparisons between the four histological groups found significant differences across the four groups except between HGMCP vs. HGMCP-SRC (p=0.624).

Multivariate analysis results are shown in Table 2. Once adjusted for other possible confounding variables (i.e. PCI, CC score, preoperative TM status … ), the PSOGI classification was no longer significantly associated to OS (p=0.870) nor to DFS (p=0.922).

**Table 2:** Multivariate analysis of risk factors of overall and disease-free survival.

	DFS HR (95 % CI)	p-Value	OSHR (95 % CI)	p-Value
**Sex**				
Male (reference)				
Female	1.060 (0.383–2.931)	0.910	0.808 (0.208–3.143)	0.880
**Age, years**	1.016 (0.970–1.064)	0.494	0.982 (0.910–1.060)	0.642
**ASA**				
1 (reference)				
2	22 (0.008–63178)	0.446	0.319 (0.035–2.887)	0.310
3	31.6 (0.015–67428)	0.377	0.345 (0.032–3.778)	0.385
**Primary vs. recurrent**	1.661 (0.75–3.68)	0.211	0	0.979
**PCI**	1.078 (1.034–1.125)	<0.001	1.048 (0.954–1.151)	0.326
**CC SCORE**				
CC0-1 (reference)				
CC2-3			3.327 (0.517–21.43)	0.206
**CEA (>5 ng/mL)**	4.34 (0.555–34.070)	0.162	1.279 (0.104–15.734)	0.847
**CA 19-9 (>23 UI/mL)**	18.66 (1.013–343)	0.049	1.626 (0.117–22.688)	0.718
**Ca-125 (>35 UI/mL)**	3.812 (0.283–51.43)	0.313	12,717 (0-4e^147^)	0.955
**Severe postoperative complications**	2.38 (0.832–6.83)	0.106	0	0.978
**PSOGI classification 1° appendiceal tumor**				
LAMN (reference)				
HAMN	0.060 (0.-2,255)	0.601	31,321 (0–5.59e^124^)	0.942
G1 ADC	1.243 (0.382–4.045)	0.988	86,247 (0–1.527e^125^)	0.936
G2-3 ADC	164 (0–2.09e^9^)	0.549	406,584 (0–7.184e^125^)	0.927
ADC w/SRC	0		2,336,971 (0–4.174e^126^)	0.917
SRCC	0		0.314 (0)	1.000
**PSOGI classification peritoneal disease**				
AM (reference)				
LGMCP	150,137 (0–1.72e^108^)	0.992	42,000 (0–1.14e^112^)	0.933
HGMCP	556,535 (0–6.37e^108^)	0.913	247,553 (0–8.51e^112^)	0.922
HGMCP-SRC	740,442 (0–8.49e^108^)	0.911	552,986 (0–1.91e^113^)	0.917
**LN**				
N0 (reference)				
N+	12.77 (0.07–23521)	0.507	0.23 (0.023–2.287)	0.210
**Postoperative SC**	0.004 (0–83326)	0.525	1.725 (0.271–10.983)	0.564

Model based on 94 cases with follow-up data for OS and 77 patients with follow-up data for DFS (patients with CC2/3 excluded), missing data handled by deletion methods. OS, overall survival; DFS, disease-free survival; ASA, American Society of Anesthesiologists Classification; SC, systemic chemotherapy; PCI, peritoneal carcinomatosis index; CC, completeness of cytoreduction; LAMN, low grade appendiceal mucinous neoplasms; G1 ADC, well-differentiated mucinous adenocarcinoma of the appendix; G2-3 ADC, moderately to poorly differentiated mucinous adenocarcinoma of the appendix; ADC w/SRC, mucinous adenocarcinoma of the appendix with signet ring cells; SRCC, signet ring cell carcinoma; PSOGI, Peritoneal Surface Oncology Group International; AM, acellular mucin; LGMCP, low grade mucinous carcinoma peritonei; HGMCP, high grade mucinous carcinoma peritonei; HGMCP-SRC, high grade mucinous carcinoma peritonei with signet ring cells; AJCC, American Joint Committee on Cancer; LN, lymph node status; HR, Hazard ratio.

### Influence of other factors on survival

Multivariable cox regression analysis was performed to study the influence of other factors on OS and DFS, results are shown in Table 2. None of the factors analyzed were significantly associated with OS. Higher PCI scores were significantly associated with shorter DFS with hazards ratio (HR) of 1.078 (1.034–1.125, p<0.001). Also, preoperative elevation of Ca19-9 was associated with shorter DFS (HR 18.66 (1.013–343), p=0.049).

## Discussion

The PSOGI consensus has helped to standardize the language around PMP and the categorization of patients with PMP. The PSOGI consensus definitions mainly relied on the association between pathological descriptions and survival outcomes from previous retrospective cohorts. In a landmark study, Ronnett et al. [4] reported a clear distinction between what they called adenomucinosis and mucinous carcinoma, and identified an intermediate prognostic group. Other important contributions to the development of the four-tiered PSOGI classification were the findings of Shetty et al. [18] and Davison et al. [19].

Since the consensus in 2016, a number of groups have aimed to evaluate whether this four-tiered histopathological classification adequately stratifies patients with regard to prognosis. This was the main objective of the present study and to compare current results with existing literature reports.

In the current series, the PSOGI classification system did distinguish between subgroups with different survival outcomes. All patients with AM peritoneal deposits were alive at 5-years, decreasing to 93.6 % in LGMCP cases, 53.6 % in HGMCP and to 66.7 % in HGMCP-SRC. These survival outcomes are in agreement with results obtained by previous study groups [8, 10, 20, 21] (see Table 3). Huang et al. [10] observed 5-year OS rates of 95.2 % in AM, 83 % in disseminated peritoneal adenomucinosis (DPAM), 47 % in peritoneal mucinous carcinomatosis (PMCA) and 12.6 % in PMCA-SRC. The nomenclature used in this study is outdated even though the pathologic subgroups correlate to those proposed by PSOGI. The results of Baratti et al. [10] showed 5-year OS rate across the different subgroups of 89.3 %, 77.5%, 51% and 0 %, respectively. However, the current series fails to demonstrate the worse survival outcomes associated with the presence of SRC [22]. This could be a chance finding due to the small total number of four in this series and patient selection bias since the four patients with SRC in this experience had optimal CRS. Similar findings were reported by a Spanish group [21].

**Table 3:** Summary of previous study groups reporting survival outcomes after cytoreductive surgery and HIPEC in PMP patients according to the PSOGI classification.

Centre [ref]	Study period	Study type	Pts, n	HIPEC regime	Histological subgroups, nº (%)	PCI med (IQR)	CC0/1, %	Morbidity, %	Mortality, %	F-up, months	OS	DFS	Px factors-OS	Px factors-DFS
National cancer institute, IT. Baratti et al., [8]	1995–2017	Retro	265	Closed	AM (26), LGPMP (197), HGPMP (38), PMP-SRC (4)	22 (12.5–28)	98.1	33.2	3.8	65.5	mOS 148.7 m 5y74.5, AM (mNR), LGPMP (m148.7), HGPMP (m63.6), SRC-PMP (m9.5), p=0.003	NA	WHO classification, PCI, CC score, preSCT	NA
				Cisplatinum (25 mL/m^2^) + MMC (3.3 mg/m^2^) 90 min										
St. George hospital, AUS. Huang et al., [10]	1996–2015	Retro	444	Open	AM (44), DPAM (232), PMCA (119), PMCA-S (49)	22 (0–39)	94.8	47.6	1.6	NA	mOS 102.5, 5y 64.5, AM (mNR), DPAM (mNR), PMCA (m58.2) PMCA-S (m31.1), p<0.001	NA	PSOGI, use of EPIC, IO transfusion, Ca19-9	NA
				LG: MMC (12.5 mg/m^2^) 90 min										
				HG: Oxali (360 mg/m^2^) 30 min.										
				LAMNs: EPIC 5FU (650 mg/m^2^)										
Reina sofia university hospital, ES. Rufian-Andujar et al., [20]	1997–2020	Retro	117	MMC (30 mg/m^2^) 60 min	AM (2.6 %), LGPMP (28.9 %), HGPMP (65.8 %), SRC-PMP (2.6 %)	21 (3–39)	93.8	16.4	3.5	47	5yOS 69.1	mDFS 24, 5y 48.3	PSOGI adjusted for CC score	PSOGI
											LGPMP (5y100), HGPMP (5y 63), SRC-PMP (not estimated), p=0.05	LGPMP (5y 77.5), HGPMP (5y 40), p=0.053		
Gregorio marañon general university hospital, ES. Martin-Roman et al., [21]	2009–2019	Retro	100	Open	AM (20), LGMCP (53), HGMCP (8), HGMCP-SRC (14)	23 (12–33)	91.5	41.4	2.1	49.2	mOS NR, 5y86.5	mDFS 64.7, 5y 51.5	PSOGI, AJCC, PCI>21	PSOGI, AJCC, postSCT, CEA, Ca19-9, Ca125
				Oxali (460 mg/m^2^) 30 min or MMC (35 mg/2) 90 min							AM (mNR), LGMCP (mNR), HGMCP (m41.4), HGMCP-SRC (m56.3), p=0.002	AM (mNR), LGMCP (m60.9), HGMCP (m13.6), HGMCP-SRC (m8.8), p<0.001		
Asan medical centre, KR. Lee et al., [23]	2007–2017	Retro	57	NA	AM (0), LGMPM (39), HGPMP (14), HGPMP-S (4)	NA	69.2	52.6^a^ (2a)	NA	50	mOS NR	mDFS NR	Postop complications CD>2a	CC score
											LGMCP (5y 56.2), HGMCP (5y 37.5), HGMCP-SRC (5y 25), p=0.001	LGMCP (5y 33.1), HGMCP 5y 15.7), HGMCP-SRC (5y 0), p=0.024.		
A. Gemelli university hospital, IT. Santullo et al., [24]	2014–2019	Retro	50	Closed	AM (10)	17 (3–34)	94	26	2	27	mOS 100, 5y 91	mDFS 77, 5y 63	CC score	CC score, histology appendiceal primary.
				First cases: Oxali	LGMCP (34)						p=0.728	p=0.001		
				MMC (35 mg/m^2^)	HGMCP (6)						OS subgroups NA.	DFS subgroups NA.		
					HGMCP-SRC (0)									
Cancer institute of the state of sao paulo ICESP, BR. Lopes et al., [25]	2008–2019	Retro	72	NA	AM (10), LGPMP (41), HGPMP (21), HGPMP-SRC (0)	NA	61	34	7	44.8	mOS 97.5	mDFS NA	NA	NA
											AM (mNR) LGPMP (m97.5), HGPMP (m86.1), p=0.15	AM (m92), LGPMP (m75.5), HGPMP (m60.9), p=0.02		
Mater misericordiae univerisity hospital, IRL (our series)	2013–2021	Retro	97	Open	AM (29), LGMCP (42), HGMCP (22), HGMCP-SRC (4)	19 (9–28)	82.7	18.3	1	30.2	mOS NR, 5yOS 88.8	mDFS NR, 5yDFS 59	None	PCI and Ca19-9
				MMC (10–12.5 mg/m^2^) 60 min							AM (5y 100), LGMCP (5y 93.7), HGMCP (5y 53.6), HGMCP-SRC (5y 66.7), p<0.001	AM (5y 100), LGMCP (5y 55.8), HGMCP (5y 18.9), HGMCP-SRC (5y 0), p<0.001		

^a^Morbidity rate for complications greater that Clavien–Dindo score >2a. NA, not available; Retro, retrospective; MMC, mitomycin C; Oxali, Oxaliplatinum; LG, low-grade; HG, high-grade; LAMNs, low-grade appendiceal mucinous neoplasm; EPIC, early postoperative intraperitoneal chemotherapy; 5-FU, 5-fluorouracil; AM, acellular mucin; LGMCP, low-grade mucinous carcinoma peritonei; HGMCP, high-grade mucinous carcinoma peritonei; HGMCP-SRC, with signet ring cells; DPAM, adenomucinosis; PMCA, peritoneal mucinous carcinomatosis; PMCA-S, with signet ring cells; PCI, peritoneal carcinomatosis index score; Med, median; IQR, interquartile range; F-up, follow-up; OS, overall survival; DFS, disease-free survival; mOS, median overall survival; mDFS, median disease-free survival; 5y, 5-year survival rates; mNR, median survival not reached; PSOGI, Peritoneal Surface Oncology Group International classification; WHO, World Health Organization classification; AJCC, 8th edition of American Joint Committee on Cancer classification; CC, Completeness of cytoreduction score; CD, Clavien-Dindo classification; postSCT, postoperative systemic chemotherapy; CEA, Carcinoembryonic antigen; Ca19-9, Carbohydrate antigen 19-9; Ca125, Cancer antigen 125.

However, on multivariate analysis adjusting for confounding variables, the predictive value of the PSOGI classification lost significance for OS (Table 2). In the available literature, the PSOGI classification significantly correlated with OS in multivariate analysis in 3 [10, 20, 21] out of 7 [8, 10, 20, 21, 23], [24], [25] studies evaluating the prognostic impact of the PSOGI classification (see Table 3). In one of these three studies [20], the AM subgroup was omitted from the PSOGI classification; therefore results should be interpreted with caution. Other factors associated with worse OS were higher PCI score [8, 21] and CC score [8], preoperative systemic chemotherapy administration [8], elevated Ca19-9 [10], intraoperative transfusion [10] and postoperative complications [23].

Similar findings were obtained regarding DFS. Significant differences were observed in the DFS rate across the different PSOGI subgroups with 5-year DFS rates of 100 % in AM; 55.8 %, in LGMCP; 18.9 %, in HGMCP and 0 % in HGMCP-SRC. However, the PSOGI classification did not predict DFS, but PCI and elevated Ca19-9 did. Five studies [20, 21, 23], [24], [25] reported the influence of the PSOGI system on DFS as well as OS (see Table 3). The PSOGI classification was significantly associated with DFS in two Spanish cohorts [20, 21]. Other identified factors were the administration of postoperative systemic chemotherapy [21] and preoperative elevation of CEA, Ca19-9 and Ca-125 [21].

In the era of molecular science and development of targeted therapies, adequate patient stratification is fundamental in defining subgroups with similar prognosis facilitating clinical decision making and enabling individualized and more efficient surveillance schemes. For example, the use of systemic chemotherapy in patients with PMP is only beneficial in those with high-grade pathological features [26]. On the opposite end of the prognostic scale, the exceptionally low recurrence rate of AM suggests that risk-adapted surveillance regime potentially limiting annual CT-scan follow up to 5 years [27]. In this respect, the capacity of the PSOGI classification to stratify patients according to survival outcomes is yet to be determined. Results from this study and the published literature are inconclusive. Nonetheless, the PSOGI classification appears to provide a better prognostic prediction when compared to other available classification systems. Two studies reported that the PSOGI classification predicted survival better than the three-tiered classification by Ronnett et al. [4, 20] and the two-tiered classification from the American Joint Committee on Cancer (AJCC) [2, 21]. On the contrary, Baratti et al. [8] concluded that the two-tiered classification proposed by the World Health Organization (WHO) [28] provided better patient stratification.

The 8th edition of the AJCC [2] and the current WHO classification updated in 2019 [29] have both incorporated the terminology and histopathological descriptions agreed upon at the PSOGI consensus. However, the 8th edition of the AJCC [2] groups together acellular mucin (M1a) and LGMCP (M1bG1) into stage IVa and HGMCP (M1bG2) and HGMCP-SRC (M1bG3) into stage IVb while patients with acellular mucin remain ungraded in the 2019 WHO classification and LGMCP, HGMCP and HGMCP-SRC are graded G1, G2 and G3 accordingly [29]. The especially low recurrence rate of patients with AM has been highlighted by multiple single-centre studies [19, 27] as has the worse prognosis associated with the presence of SRC [22, 30]. As a result, the four-tiers proposed by the PSOGI classification [7] seem logical even though only evidence of low quality can support the current use of this classification.

The limitations of this study are its retrospective design and small sample, particularly with one group consisting of only 4 patients.

In conclusion, we report survival outcomes after CRS + HIPEC treatment for PMP patients in a national referral centre. The results have been analyzed after categorization into the PSOGI Pathology Consensus system. In univariate analysis, the PSOGI system predicts OS and DFS but this significance is lost in multivariate analysis where the extent of disease seems to be the main predictor of outcome. Current histological classification alone does not correlate accurately with outcome.
